# Magnetic resonance imaging volumetry in atypical parkinsonism: current evidence and neuroradiological perspectives

**DOI:** 10.3389/fnagi.2026.1816084

**Published:** 2026-04-21

**Authors:** Bartosz Migda, Natalia Madetko-Alster, Anna Migda, Michał Kutyłowski, Piotr Alster

**Affiliations:** 1 Diagnostic Ultrasound Lab, Department of Pediatric Radiology, Medical University of Warsaw, Warsaw, Poland; 2 Department of Neurology, Medical University of Warsaw, Warsaw, Poland; 3 Department of Endocrinology, Diabetology and Internal Medicine, Medical University of Warsaw, Warsaw, Poland; 4 Department of Radiology, Mazovian Brodno Hospital, Warsaw, Poland

**Keywords:** atypical parkinsonism, brainstem atrophy, corticobasal syndrome, MRI volumetry, MRPI 2.0, multiple system atrophy, neurodegeneration, progressive supranuclear palsy

## Abstract

Atypical parkinsonism, including progressive supranuclear palsy (PSP), multiple system atrophy (MSA), and corticobasal syndrome (CBS), represents a major diagnostic challenge, particularly in the early stages of disease. Magnetic resonance imaging (MRI) plays a pivotal role in the differential diagnosis of these disorders. Beyond conventional qualitative assessment, quantitative MRI techniques, especially volumetry and planimetry, have gained increasing importance as objective tools for evaluating disease specific patterns of brain atrophy. This narrative review summarizes current evidence on MRI-based morphometric approaches in atypical parkinsonism, with a particular focus on brainstem, basal ganglia, cerebellar, and cortical measurements. The diagnostic value of established indices, including the Magnetic Resonance Parkinsonism Index (MRPI) and its updated version, MRPI 2.0, is discussed, along with advances in automated segmentation and machine learning based approaches. The potential role of MRI volumetry as both a diagnostic and progression biomarker is highlighted, together with current limitations and future perspectives relevant to neuroradiological practice.

## 1 Introduction

Atypical parkinsonism, encompassing progressive supranuclear palsy (PSP), multiple system atrophy (MSA), and corticobasal syndrome (CBS), represents a well-recognized diagnostic challenge in both clinical and neuroradiological practice. In the early stages of disease, these disorders frequently present with motor and non-motor features that overlap with idiopathic Parkinson’s disease, including bradykinesia, rigidity, and postural instability. Moreover, the response to levodopa therapy is typically absent or only modest, and rarely sustained, limiting its value as a reliable clinical discriminator and often delaying accurate diagnosis (Hoglinger et al., 2017; Tolosa et al., 2006; Williams & Litvan, 2013).

Magnetic resonance imaging (MRI) plays a central role in the differential diagnosis of atypical parkinsonism by enabling the identification of disease specific patterns of brain atrophy. In addition to conventional qualitative assessment, quantitative MRI techniques particularly volumetry and planimetry have gained increasing attention over the past two decades as objective tools for structural analysis. Previous studies have demonstrated that quantitative measurements of the brainstem, cerebellum, basal ganglia, and cerebral cortex provide reproducible markers of neurodegeneration and improve diagnostic confidence compared with visual assessment alone, while reducing observer-dependent variability (Boxer et al., 2006; Price et al., 2004; Schulz et al., 1999).

Brainstem morphometric analysis occupies a central position among quantitative MRI techniques used in atypical parkinsonism. In progressive supranuclear palsy (PSP), pronounced midbrain atrophy with relative preservation of pontine volume represents a well-established and reproducible imaging hallmark. This characteristic pattern has been incorporated into simple planimetric measures, such as the midbrain-to-pons ratio, as well as into more complex composite indices, most notably the Magnetic Resonance Parkinsonism Index (MRPI), which combines multiple brainstem and cerebellar peduncle measurements to improve diagnostic accuracy (Quattrone et al., 2008; Massey et al., 2013). In contrast, multiple system atrophy (MSA) is characterized by a distinct pattern of volumetric changes predominantly involving the pons, cerebellum, and basal ganglia, particularly the putamen. These alterations reflect the underlying multisystem neurodegenerative process and differ substantially from the midbrain-predominant atrophy observed in PSP. In the parkinsonian variant of MSA (MSA-P), volumetric reduction of the putamen represents a key imaging feature, whereas in the cerebellar variant (MSA-C), pronounced atrophy of the cerebellum and pontine structures predominates. Quantitative MRI studies have demonstrated that volumetric assessment of these regions improves differentiation of MSA from both PSP and Parkinson’s disease, especially when combined with multiregional or automated volumetric approaches (Huppertz et al., 2016; Sjöström et al., 2020).

Consistent with these imaging characteristics, current diagnostic criteria for atypical parkinsonian syndromes incorporate neuroimaging with varying weight depending on the disease entity. In the Movement Disorder Society (MDS) criteria for progressive supranuclear palsy, MRI findings are considered supportive, reflecting characteristic patterns of midbrain atrophy and related brainstem involvement, but imaging features are not mandatory for diagnostic categorization. These criteria emphasize a predominantly clinical, phenotype-based approach, with MRI serving to increase diagnostic confidence, particularly in clinically ambiguous cases (Hoglinger et al., 2017).

In contrast, the MDS criteria for multiple system atrophy assign a more formal role to neuroimaging. For the category of clinically established MSA, the presence of specific MRI markers reflecting pontocerebellar and/or putaminal involvement is required, whereas in clinically probable MSA imaging findings are considered supportive (Wenning et al., 2022). This distinction underscores the central role of MRI in confirming the diagnosis of MSA and highlights its value beyond clinical assessment alone.

For corticobasal syndrome, which represents a clinical syndrome rather than a single neuropathological entity, neuroimaging is primarily supportive and exclusionary. In commonly used diagnostic criteria for corticobasal degeneration, MRI contributes to the identification of asymmetric cortical and subcortical atrophy patterns typical of CBS and aids in excluding alternative etiologies, but imaging findings are not diagnostic in isolation (Armstrong et al., 2013). Overall, these criteria reflect a graded incorporation of neuroimaging, ranging from supportive use in PSP and CBS to a mandatory role in specific diagnostic categories of MSA.

Recent advances in automated segmentation, atlas based volumetry, and machine learning algorithms have substantially expanded the diagnostic potential of MRI in atypical parkinsonism. Automated whole-brain and brainstem volumetric approaches enable objective, multiregional assessment of neurodegeneration and reduce observer dependency inherent to manual measurements. Previous studies have demonstrated that automated volumetry, particularly when combined with multivariate classification models, achieves promising diagnostic accuracy in differentiating progressive supranuclear palsy, multiple system atrophy, and Parkinson’s disease. Moreover, disease specific patterns of brain atrophy identified through automated analyses including characteristic brainstem involvement in PSP, pontocerebellar atrophy in MSA, and asymmetric cortical degeneration in corticobasal syndrome support the role of these techniques in both differential diagnosis and phenotypic characterization (Brinia et al., 2023; Kawabata et al., 2024; Krismer et al., 2024). Importantly, MRI volumetry is increasingly regarded not only as a diagnostic tool but also as a potential biomarker of disease progression and a quantitative outcome measure in clinical trials. This concept is particularly well supported in multiple system atrophy (MSA), where longitudinal MRI studies have demonstrated consistent and measurable rates of atrophy involving the pons, cerebellum, and basal ganglia. Multicenter investigations have shown that volumetric MRI metrics exhibit larger effect sizes and greater sensitivity to disease-related change over time than conventional clinical scales, highlighting their potential utility for monitoring disease progression and evaluating therapeutic interventions (Brinia et al., 2023; Krismer et al., 2024; Yekhlef et al., 2003; Wenning et al., 1997). Importantly, a growing body of evidence indicates that this framework is equally applicable to progressive supranuclear palsy (PSP) and related tauopathies. Longitudinal MRI studies have demonstrated that regional brain atrophy can be reliably quantified over relatively short intervals in PSP, with measurable volume loss detectable within 6–12 months and correlating with clinical progression, as shown by Dutt et al. (2016). More recent large-scale and multicenter analyses further support the role of MRI-based measures as progression biomarkers in PSP. Quattrone et al. (2024) demonstrated that longitudinal changes in brainstem and multiregional volumetric metrics exhibit high effect sizes and allow detection of treatment effects with relatively small sample sizes, underscoring their value as imaging endpoints in clinical trials. Similarly, Kumar et al. (2025) showed that region-specific atrophy rates differ across PSP clinical variants and that combining volumetric measures with clinical scales provides optimal statistical power for trial design. These findings are consistent with results from the PROSPECT-M-UK study, in which neuroimaging metrics outperformed conventional clinical measures in terms of sensitivity to change and sample size requirements across atypical parkinsonian syndromes, including PSP, MSA, and CBS (Street et al., 2023).

The aim of this narrative review is to provide a comprehensive overview of the current evidence regarding MRI volumetry in atypical parkinsonism, emphasizing its diagnostic utility, differentiating value, and potential role as a disease biomarker from a neuroradiological perspective.

## 2 Literature review methods

This review was conducted as a narrative literature review. Publications from 1995 to 2024 were considered, allowing inclusion of both seminal studies introducing volumetric MRI techniques and more recent investigations employing automated segmentation and machine learning approaches.

The literature search was performed using PubMed/MEDLINE, Scopus, and Web of Science databases. Search strategies were based on expert-driven combinations of the following keywords: “MRI volumetry,” “brain volumetry,” “brainstem volumetry,” “planimetry,” “atypical parkinsonism,” “progressive supranuclear palsy”, “multiple system atrophy”, “corticobasal syndrome”, “MRPI”, and “MRPI 2.0.”

Eligible publications included original research articles, prospective and retrospective studies, longitudinal studies, methodological papers, as well as systematic reviews and meta-analyses. Only studies published in English and involving human subjects were included.

Inclusion criteria were:

(1) Application of MRI volumetric or planimetric techniques.
(2) Analysis of patients with PSP, MSA, or CBS.
(3) Relevance to differential diagnosis, disease progression, or clinical application.

Exclusion criteria were:

(1) Single case reports without quantitative analysis.
(2) Studies relying solely on qualitative MRI assessment.
(3) Studies focusing exclusively on Parkinson’s disease without comparison to atypical parkinsonism.

## 3 MRI Morphometric Techniques

### 3.1 Manual and semi-automated planimetry

Early quantitative MRI approaches in atypical parkinsonism predominantly relied on manual or semi automated planimetric measurements of selected brainstem structures, typically performed on midsagittal T1-weighted images. These methods focused on regions known to be differentially affected by distinct parkinsonian syndromes, particularly the midbrain, pons, and cerebellar peduncles.

Seminal work by Quattrone et al. (2008) introduced the Magnetic Resonance Parkinsonism Index (MRPI), a composite planimetric measure integrating the pons-to-midbrain area ratio and the middle cerebellar peduncle-to-superior cerebellar peduncle width ratio. By combining measurements of four brainstem structures with distinct patterns of involvement in progressive supranuclear palsy and multiple system atrophy, MRPI demonstrated excellent diagnostic performance for differentiating PSP from Parkinson’s disease and the parkinsonian variant of MSA, even at the individual patient level. Complementary to this composite approach, Massey et al. (2013) proposed a simplified midbrain-to-pons ratio, derived from linear measurements on midsagittal MRI. Importantly, this marker was validated in pathologically confirmed cases, demonstrating high specificity for PSP and excellent interrater reliability. The study highlighted that simple manual measurements, when grounded in disease specific patterns of neurodegeneration, can outperform purely qualitative MRI signs and remain feasible for routine clinical use.

While manual and semi-automated planimetric techniques are relatively easy to implement in routine clinical practice, their main limitations include observer dependency and inter-rater variability. As highlighted by (Krismer & Seppi, 2018), early region-of-interest based measurements of brainstem structures are sensitive to differences in ROI placement, slice selection, and disease stage, which may result in overlapping individual values and variability in diagnostic performance across studies. These methodological constraints have contributed to inconsistent results among planimetric indices and provided a strong rationale for the development of more robust and standardized composite measures, such as MRPI 2.0. Importantly, this technical variability reflects underlying biological heterogeneity. Large clinicopathological series by Wenning et al. (1997) demonstrated marked interindividual variability in the severity and distribution of olivopontocerebellar and striatonigral degeneration, even among pathologically confirmed cases. Such heterogeneity, together with frequent overlap between affected and relatively preserved brainstem regions, inherently limits the precision of two-dimensional manual measurements and further contributes to observer dependent variability. These limitations provided a strong rationale for the development of more standardized composite indices and automated volumetric approaches.

### 3.2 Atlas based volumetry

The limitations of manual and semi-automated planimetric techniques have driven the development of more comprehensive volumetric approaches based on automated segmentation and anatomical atlases. As highlighted by (Krismer & Seppi, 2018), region-of-interest based measurements are inherently constrained by observer dependency, overlap of individual values, and limited representation of the spatial complexity of neurodegenerative processes. These shortcomings restrict their ability to fully capture disease specific atrophy patterns, particularly in clinically heterogeneous syndromes and early disease stages.

Importantly, neuropathological evidence further supports the need for multiregional volumetric assessment. Large clinicopathological series by Wenning et al. (1997) demonstrated marked interindividual variability in the distribution and severity of neurodegeneration across brainstem, cerebellar, and basal ganglia structures, even among pathologically confirmed cases of atypical parkinsonism. This biological heterogeneity limits the interpretability of single-structure measurements and underscores the necessity for methods capable of assessing the entire brain in a standardized manner.

Atlas based volumetric techniques address these challenges by enabling automated and observer independent quantification of multiple brain regions. By leveraging predefined anatomical atlases and standardized segmentation pipelines, these approaches allow objective assessment of brainstem, basal ganglia, cerebellar, and cortical volumes, providing a more comprehensive overview of disease related atrophy patterns in atypical parkinsonism. From a neuroradiological perspective, atlas based volumetry represents a critical methodological advancement that bridges the gap between focal planimetric indices and fully automated, data driven classification models.

Early methodological foundations were established by voxel based morphometry (VBM), which combines spatial normalization, tissue segmentation, and voxel-wise statistical analysis to assess regional gray matter differences on a whole-brain level. Introduced by (Ashburner & Friston, 2000), VBM enabled unbiased, automated assessment of structural brain changes without *a priori* region selection, laying the groundwork for subsequent atlas based volumetric approaches. Further developments in diffeomorphic and high-dimensional registration algorithms improved anatomical correspondence between subjects, enhancing sensitivity to subtle disease related atrophy patterns (Ashburner, 2007).

Surface based and volumetric atlas approaches were subsequently refined through the development of automated segmentation frameworks such as FreeSurfer, which integrates intensity normalization, topology correction, and probabilistic atlas information to provide robust measurements of cortical thickness, surface area, and subcortical volumes. Validation studies demonstrated high anatomical accuracy and excellent intra- and inter rater reliability, supporting the use of atlas-based segmentation for longitudinal and multicenter studies (Fischl, 2012). The introduction of standardized cortical parcellation schemes, including the Desikan atlas, further facilitated harmonized regional analyses across studies and disease populations (Desikan et al., 2006).

Complementary atlas based approaches have focused on subcortical and brainstem structures. The FSL-FIRST framework employs Bayesian shape and appearance models trained on manually labeled datasets to achieve automated, anatomically constrained segmentation of basal ganglia, thalamus, and brainstem structures. This methodology allows not only volumetric assessment but also vertex-wise shape analysis, enabling localization of disease related structural changes beyond global volume loss (Patenaude et al., 2011).

Collectively, atlas based volumetric techniques provide a comprehensive and standardized framework for quantifying regional brain atrophy, encompassing cortical, subcortical, cerebellar, and brainstem structures. In the context of atypical parkinsonism, such approaches are particularly well suited to capture the spatial complexity and biological heterogeneity of neurodegenerative processes, overcoming the intrinsic limitations of focal planimetric measurements and enabling integration with advanced statistical and machine learning models.

### 3.3 Automated volumetry and machine learning

Recent advances in fully automated volumetric analysis have increasingly been combined with machine learning techniques to enhance the differential diagnosis of atypical parkinsonian syndromes at the individual patient level. Unlike conventional region based approaches, machine learning frameworks integrate multidimensional volumetric features and capture inter-regional relationships that are not apparent in univariate analyses. However, the incremental diagnostic gain of complex machine learning models over estabilished semi-quantitative indices such as MRPI remains incompletely estabilished in real-world cohorts.

Early applications of supervised machine learning demonstrated the feasibility of whole-brain MRI based classification in parkinsonian disorders. Salvatore et al. (2014) employed a combination of principal component analysis for feature extraction and support vector machines for classification, achieving high diagnostic accuracy in differentiating progressive supranuclear palsy from Parkinson’s disease on an individual basis. Importantly, this approach did not rely on predefined regions of interest, instead identifying distributed voxel wise morphological patterns involving the midbrain, pons, thalamus, and corpus callosum structures known to be critically affected in PSP.

Subsequent studies expanded these concepts by integrating fully automated segmentation pipelines with multiregional volumetric features and conventional machine learning classifiers, including support vector machines and random forest algorithms. These approaches enabled simultaneous assessment of cortical, subcortical, cerebellar, and brainstem volumes, demonstrating promising diagnostic performance in differentiating PSP, MSA, and corticobasal syndrome. However, despite their high accuracy, these methods typically require specialized image preprocessing, feature selection, and expertise in computational analysis, limiting their implementation to research-oriented or specialized centers (Brinia et al., 2023).

More recently, deep learning–based frameworks have been introduced, allowing end to end classification directly from structural MRI data with minimal manual feature engineering. Convolutional neural network models have shown further improvements in diagnostic accuracy and robustness across parkinsonian phenotypes. Nevertheless, challenges related to interpretability, generalizability across scanners and centers, and the lack of standardized validation pipelines currently constrain their routine clinical adoption (Nigro et al., 2024).

These limitations are further emphasized by methodological considerations related to model development and validation. A key issue highlighted in the literature is the limitation related to sample size. As emphasized by (Varoquaux, 2018), many neuroimaging studies operate on relatively small cohorts, which inherently leads to large uncertainty in model performance estimates. Even with cross-validation, error margins can be substantial, and commonly reported accuracy metrics may therefore be overly optimistic and not sufficiently reliable for clinical interpretation.

Closely related to this is the risk of overfitting. Wen et al. (2020) in a comprehensive evaluation of deep learning models in neuroimaging, demonstrated that a significant proportion of published studies may report biased performance due to methodological issues such as data leakage or inadequate validation strategies. Importantly, they showed that models often perform well within a given dataset but fail to generalize to independent cohorts with different demographic or clinical characteristics.

The need for rigorous validation is further underscored by Poldrack et al. (2020) who outline best practices for predictive modeling in neuroimaging. They emphasize that true predictive performance must be assessed on independent data not used during model development, and that cross-validation procedures must encompass the entire analytical pipeline. Notably, they caution against the use of small samples and recommend large datasets and multiple performance metrics to ensure robust and generalizable findings.

Another critical aspect is the mismatch between experimental validation and real-world clinical application. As discussed by Saeb et al. (2017) validation strategies must reflect the intended clinical use-case. In particular, subject-level validation is essential, as alternative approaches may substantially overestimate model performance and fail to represent real diagnostic scenarios.

Furthermore, generalizability across cohorts remains a major challenge. Dinsdale et al. (2021) demonstrated that even in large datasets, model performance may be influenced by preprocessing steps and dataset-specific characteristics, with networks potentially learning artifacts rather than biologically meaningful patterns. This raises concerns regarding the robustness of models when applied to external datasets.

From a broader clinical perspective, Kelly et al. (2019) highlight that machine learning systems are particularly vulnerable to issues such as dataset shift, confounding factors, and limited generalizability, all of which may hinder their safe implementation in clinical workflows. They stress the importance of external validation on independent and representative datasets, as well as the need for models to demonstrate real clinical utility beyond technical accuracy.

Importantly, these challenges are further compounded by the intrinsic limitations of the clinical reference standard in atypical parkinsonian syndromes. A definitive diagnosis can be established with certainty only through neuropathological examination, while *in vivo* diagnoses remain probabilistic. In addition, certain clinical entities, particularly corticobasal syndrome (CBS), are associated with heterogeneous underlying pathologies, which introduces additional variability and uncertainty in model training and validation datasets. This further limits the interpretability and generalizability of machine learning models across cohorts.

Taken together, these findings indicate that, despite promising results, machine learning approaches in MRI analysis remain subject to important methodological constraints. At present, these methods should be interpreted with caution and are best considered complementary tools within research or specialized settings, rather than fully established components of routine clinical decision-making.

Automated volumetry combined with machine learning represents a powerful extension of quantitative MRI, enabling characterization of the spatial complexity and biological heterogeneity of atypical parkinsonism. Although these techniques currently remain largely confined to research settings, ongoing methodological standardization and external validation may facilitate their gradual integration into routine clinical workflows.

Table 1 provides a concise overview of disease-specific volumetric patterns, key quantitative MRI markers, and their principal clinical applications across atypical parkinsonian syndromes.

**Table 1:** Summary of MRI volumetric patterns and quantitative markers in atypical parkinsonian syndromes.

Disorder	Predominant volumnetric pattern	Key quantitative MRI markers	Clinical utility	Main limitations
PSP	Marked midbrain atrophy with relative preservation of pontine volume, mostpronounced in the Richardson syndrome phenotype (Oba et al., 2005)	Midbrain area, midbrain-to-pons ratio, MRPI, MRPI 2.0; additional phenotype-associated findings include hippocampal atrophy (predominantly in PSP-RS) and corpus callosum involvement identified in whole-brain volumetric and voxel-based analyses (Quattrone et al., 2008; Quattrone et al., 2020)	High diagnostic specificity for PSP-RS; useful for differentiation from PD and MSA (Massey et al., 2013)	Reduced sensitivity in non-Richardson PSP variants; limited coverage of supratentorial involvement (Hoglinger et al., 2017)
MSA	Pontocerebellar and putaminal atrophy reflecting oligo pontocerebellar and striatonigral degeneration (Wenning et al., 2013)	Pontine and cerebellar volumes, putaminal volume, regional atrophy rates (Paviour et al., 2006)	Differentiation of MSA phenotypes; robust longitudinal marker of disease progression (Paviour et al., 2006)	Overlap with other atypical parkinsonian syndromes in early disease; dependence on acquisition protocols (Meissner et al., 2019)
CBS	Asymmetric frontoparietal cortical atrophy with variable subcortical involvement, depending on underlying pathology (Whitwell et al., 2010)	Asymmetric cortical volumes, VBM-derived gray and white matter changes (Whitwell et al., 2010; Borroni et al., 2015)	Supportive for syndrome recognition; may aid etiological inference (e.g., AD vs. FTLD) (Whitwell et al., 2010)	Lack of pathology specificity; limited value of brainstem-focused markers (Armstrong et al., 2013)
All syndromes	Multiregional patterns reflecting heterogeneous neurodegeneration (Huppertz et al., 2016)	Atlas based whole brain volumetry; multiregional feature sets (Ashburner & Friston, 2000; Fischl, 2012)	Objective and reproducible assessment; suitable for research and multicenter studies (Salvatore et al., 2014)	Limited availability in routine practice; lack of harmonized normative datasets (Fortin et al., 2018; Pomponio et al., 2020)

Table summarizes key volumetric patterns and quantitative MRI markers based on evidence synthesized from the cited literature.

## 4 MRI volumetry in progressive supranuclear palsy

Progressive supranuclear palsy (PSP) comprises a clinically and pathologically heterogeneous group of phenotypes, within which the Richardson syndrome (PSP-RS) represents the most typical and best-characterized variant. PSP-RS is associated with a distinctive and highly reproducible pattern of brainstem atrophy, dominated by pronounced midbrain volume loss with relative preservation of pontine volume. Among atypical parkinsonian syndromes, this morphometric signature constitutes the most consistent and well documented MRI finding and forms the basis for several quantitative diagnostic indices. However, it should be emphasized that in non-Richardson PSP phenotypes, including PSP-parkinsonism (PSP-P) and other variant presentations, brainstem atrophy may be less pronounced or even absent in early disease stages, limiting the sensitivity of purely brainstem-based markers (Hoglinger et al., 2017; Whitwell et al., 2010).

Early quantitative MRI studies demonstrated that midbrain area measurements provide robust discrimination between PSP and other parkinsonian disorders. (Massey et al., 2013) showed that midbrain atrophy is markedly more severe in PSP than in Parkinson’s disease, translating the visually recognized “hummingbird sign” into objective, reproducible planimetric metrics. These observations were corroborated by subsequent studies confirming that midbrain measurements yield high specificity even in clinically ambiguous cases Oba et al., (2005).

Building on these findings, Quattrone et al. (2008) introduced composite indices integrating midbrain and pontine measurements, most notably the midbrain-to-pons ratio and the Magnetic Resonance Parkinsonism Index (MRPI). These indices demonstrated excellent diagnostic performance in differentiating PSP from Parkinson’s disease and multiple system atrophy, with particularly high specificity for the PSP Richardson syndrome variant, in which axial rigidity, vertical gaze palsy, and early postural instability are most prominent.

Volumetric involvement in PSP extends beyond the midbrain. Quantitative MRI studies have consistently demonstrated atrophy of the superior cerebellar peduncle, reflecting degeneration of the dentato-rubro-thalamic tract. Paviour et al. (2005) showed that superior cerebellar peduncle volume is significantly reduced in PSP compared with Parkinson’s disease and MSA, supporting its role as a complementary biomarker that enhances diagnostic confidence when combined with midbrain measurements. In addition, in PSP-RS, quantitative MRI and voxel-based morphometry studies have demonstrated concomitant atrophy of limbic structures, including the hippocampus, suggesting more widespread neurodegeneration extending beyond the brainstem (Whitwell et al., 2007; Josephs et al., 2008).

Advanced voxel based morphometry studies have further validated these region specific findings at the whole brain level. Huppertz et al. (2016) demonstrated that PSP is characterized by prominent white matter loss in the mesencephalon and adjacent brainstem structures, and that these volumetric patterns can be leveraged for accurate individual level classification using machine learning techniques (Focke et al., 2011). Notably, whole brain analyses in PSP have also identified involvement of commissural pathways, particularly the corpus callosum, reflecting disruption of interhemispheric connectivity in advanced and variant phenotypes (Whitwell et al., 2011).

Importantly, meta-analytic evidence has confirmed that midbrain area based metrics and MRPI yield among the largest diagnostic effect sizes of all MRI parameters evaluated in PSP, outperforming many cortical and subcortical volumetric measures. Nevertheless, subgroup analyses indicate that these effect sizes are driven predominantly by PSP-RS cohorts, underscoring the reduced sensitivity of brainstem-focused metrics in variant PSP phenotypes (Krismer & Seppi, 2018; Mattia et al., 2025).

MRI volumetry in PSP therefore exemplifies how targeted quantitative assessment of disease specific neuroanatomical vulnerability can translate into clinically meaningful biomarkers, while also highlighting the importance of phenotype aware and multiregional interpretation when applying volumetric indices across the PSP spectrum.

### 4.1 MRPI 2.0

MRPI 2.0 represents a refined extension of the classical Magnetic Resonance Parkinsonism Index, designed to address the reduced sensitivity of brainstem based markers in specific PSP phenotypes, particularly PSP parkinsonism (PSP-P). In addition to the original MRPI components midbrain and pons areas and the widths of the superior and middle cerebellar peduncles MRPI 2.0 incorporates measurements of the third ventricle width normalized to the frontal horn width of the lateral ventricles. These additional parameters capture disease related periventricular atrophy that is frequently observed in PSP but may be insufficiently reflected by brainstem measurements alone (Quattrone et al., 2018).

The rationale for this modification stems from clinical and imaging observations indicating that PSP-P often exhibits less pronounced midbrain atrophy in the early disease stages compared with the Richardson syndrome variant, resulting in reduced diagnostic sensitivity of the classical MRPI. By integrating ventricular measurements, MRPI 2.0 enhances sensitivity for PSP-P while preserving the high specificity that characterizes brainstem-based indices (Quattrone et al., 2018; Schocke et al., 2002).

Subsequent large scale studies have confirmed the diagnostic advantages of MRPI 2.0. A comprehensive development and validation study demonstrated that automated MRPI 2.0 significantly outperformed the classical MRPI in differentiating PSP-P from Parkinson’s disease, particularly in early stage patients, while maintaining excellent specificity. Importantly, the automated implementation ensured high reproducibility and minimized observer dependency, supporting its applicability across centers (Quattrone et al., 2023).

Independent external validation further supported the clinical utility of MRPI 2.0 in selected diagnostic scenarios. Madetko et al. (2022) evaluated MRPI 2.0 in a real world clinical cohort including patients with PSP, other atypical parkinsonian syndromes, and healthy controls, and demonstrated that MRPI 2.0 effectively discriminates PSP, including PSP-P, from healthy individuals. However, the study also showed that MRPI 2.0 does not consistently outperform classical MRPI or the midbrain-to-pons ratio in differentiating PSP-P from other atypical parkinsonian syndromes, particularly MSA-P. The authors suggested that incorporation of ventricular measurements may improve sensitivity for PSP-P by capturing periventricular changes not fully reflected by brainstem-based indices alone.

From a neuroradiological perspective, MRPI 2.0 represents a robust, reproducible, and clinically feasible quantitative index that can be readily derived from routine 3D T1-weighted MRI without the need for advanced post-processing or machine learning pipelines. In cases with ambiguous clinical presentation or atypical disease evolution, MRPI 2.0 serves as a valuable extension of standard morphological MRI assessment, substantially improving diagnostic confidence, particularly in the challenging differentiation of PSP-P from Parkinson’s disease.

## 5 MRI volumetry in multiple system atrophy

Multiple system atrophy (MSA) is characterized by a distinct pattern of neurodegeneration predominantly affecting infratentorial structures and the basal ganglia, with volumetric MRI providing valuable insights into both phenotypic differentiation and disease progression. Unlike progressive supranuclear palsy, in which midbrain atrophy predominates, MSA demonstrates preferential involvement of the pons, cerebellum, and putamen, reflecting the underlying olivopontocerebellar and striatonigral pathology (Schocke et al., 2002).

Volumetric patterns differ substantially between the two main clinical phenotypes. In the parkinsonian variant (MSA-P), quantitative MRI studies consistently demonstrate putaminal volume reduction, frequently accompanied by characteristic signal abnormalities, including a hyperintense rim and hypointensity on T2-weighted images. These supratentorial changes provide important differentiating features from Parkinson’s disease, particularly in early disease stages when clinical overlap is common. In contrast, the cerebellar variant (MSA-C) is dominated by marked cerebellar and pontine atrophy, often involving the middle cerebellar peduncles, reflecting degeneration of ponto-cerebellar pathways (Lee et al., 2004).

Beyond cross-sectional discrimination, longitudinal volumetric MRI has emerged as a particularly powerful tool in MSA, offering objective markers of disease progression. In a seminal longitudinal study, Paviour et al. (2006) demonstrated that patients with MSA-P exhibit the highest annualized rates of pontine and cerebellar atrophy among parkinsonian disorders, exceeding both PSP and Parkinson’s disease. Importantly, these regional atrophy rates were substantially greater than whole brain atrophy rates and showed lower variability, underscoring their robustness as quantitative biomarkers.

Clinico radiological correlations further support the relevance of volumetric measures in MSA. Accelerated ponto-cerebellar atrophy rates have been shown to correlate with worsening motor disability and cognitive decline, indicating that regional volumetric changes reflect clinically meaningful disease progression rather than nonspecific neurodegeneration (Paviour et al., 2006). These findings highlight the potential utility of MRI-derived atrophy rates as outcome measures in therapeutic trials, particularly in the context of emerging disease-modifying strategies for MSA.

MRI volumetry in MSA provides both diagnostic and prognostic value by enabling differentiation between disease phenotypes and offering quantitative markers of disease progression. Compared with static cross-sectional measurements, longitudinal assessment of regional atrophy, particularly in the pons, cerebellum, and putamen, appears especially well suited for monitoring disease evolution and evaluating treatment effects in clinical research settings.

## 6 MRI volumetry in corticobasal syndrome

Corticobasal syndrome (CBS) should be regarded as a clinical syndrome rather than a single neuropathological entity, and is typically associated with an asymmetric pattern of cortical and subcortical neurodegeneration. This asymmetry constitutes a key imaging feature distinguishing CBS from the more brainstem predominant phenotypes of PSP and the ponto-cerebellar/striatal patterns of MSA. Importantly, CBS may arise from different underlying pathologies, including corticobasal degeneration (CBD), Alzheimer’s disease (AD), frontotemporal lobar degeneration (FTLD-TDP), and, less frequently, PSP, which has important implications for the interpretation of neuroimaging findings (Armstrong et al., 2013; Whitwell et al., 2010).

Quantitative MRI particularly voxel-based morphometry (VBM) and atlas based volumetry has consistently demonstrated that CBS is characterized by asymmetric volume loss predominantly involving frontoparietal regions, with variable involvement of the basal ganglia and thalamus. However, the precise topography and extent of atrophy differ according to the underlying pathology, reflecting the biological heterogeneity that underlies the CBS phenotype.

A major neuroradiological challenge in CBS is therefore the lack of a one to one correspondence between the clinical syndrome and neuropathology. In an autopsy confirmed cohort, Whitwell et al. (2010) showed that atrophy patterns in patients presenting with CBS differ depending on the underlying pathology, with certain regions being commonly affected across CBS variants (premotor cortex, supplementary motor area, and insula), while more widespread frontotemporal atrophy may suggest FTLD-TDP and predominant temporoparietal involvement (including the parietal lobe and precuneus) may point toward Alzheimer-type pathology presenting as CBS. These findings indicate that quantitative MRI in CBS may contribute not only to syndrome recognition but also to etiological inference, which is increasingly relevant for biomarker-driven trial stratification.

Importantly, quantitative MRI changes may be detectable early, when clinical differentiation from idiopathic Parkinson’s disease (PD) remains difficult. Ferrea et al. (2022) demonstrated that VBM can identify disease specific gray and white matter volume differences between CBS and PD as early as approximately 1.5–2 years after symptom onset, with prominent frontoparietal white matter involvement and additional cortical changes. These findings support the potential role of automated morphometric approaches as an adjunct in early differential diagnosis in clinically ambiguous cases.

While brainstem planimetry and indices such as MRPI are central in PSP, brainstem volumetry plays a limited role in CBS, as the dominant signal of neurodegeneration is typically cortical and network-based rather than confined to mesencephalic structures. Accordingly, cortical volumetry and VBM provide more clinically relevant information in CBS, particularly when combined with assessments of white matter integrity and clinico-anatomical correlations. In an early stage CBDS cohort, Borroni et al. (2008) reported asymmetric cortical gray matter loss (with prominent frontal and parietal involvement) and demonstrated that structural changes especially within parietal regions and frontoparietal connecting pathways correlate with limb apraxia. This reinforces the concept that quantitative MRI captures clinically meaningful network disruption rather than isolated regional atrophy in CBS.

From a practical standpoint, volumetric assessment in CBS is therefore most informative when interpreted within a syndromic etiological framework, focusing on asymmetric cortical patterns (frontoparietal and premotor/SMA regions), with supportive evaluation of subcortical structures and the thalamus and where available quantitative white matter metrics. Such an approach may improve diagnostic confidence, facilitate early differentiation from PD, and support etiological stratification (e.g., CBS-AD vs. CBS-FTLD vs. CBS-CBD), which is increasingly important in the era of pathology-specific therapeutic trials.

## 7 Discussion

### 7.1 Clinical value and current position of MRI volumetry

MRI-based morphometry in atypical parkinsonian syndrome has evolved from a descriptive structural tool to a potential biomarker framework. However, significant conceptual, methodological and translational challenges remain. From a neuroradiological perspective, MRI volumetry constitutes a valuable extension of conventional morphological assessment by enabling objective quantification of regional atrophy and facilitating more standardized comparisons across patients and groups. From a clinical standpoint, the integration of volumetric MRI into routine practice remains uneven, reflecting the gap between methodological advances and real-world availability. In multicenter research settings, atlas based volumetry pipelines implemented within established neuroimaging frameworks (e.g., SPM based segmentation/normalization) allow extraction of regional tissue volumes and intracranial volume measures in a structured, reproducible manner, thereby supporting both diagnostic research and longitudinal monitoring Huppertz et al. (2016).

Based on the current literature, including both large validation studies and longitudinal analyses, MRPI, MRPI 2.0, and volumetric MRI measures are consistently supported as reliable diagnostic biomarkers, particularly for differentiating progressive supranuclear palsy (PSP) from Parkinson’s disease. For example, studies by Quattrone et al. (2022) demonstrate that MRPI 2.0 provides high diagnostic accuracy and reproducibility, including in early-stage disease, and may assist in patient stratification and clinical trial selection. Similarly, longitudinal work by Wang et al. (2025) highlights that MRPI-derived metrics correlate with disease progression, supporting their role as potential progression biomarkers.

However, despite this robust diagnostic performance, these studies predominantly focus on sensitivity, specificity, and group-level discrimination, rather than on real-world clinical implementation. As noted in multiple analyses, including those by Madetko et al. (2022); Mostile et al., 2016) there is a lack of standardized recommendations defining when these measures should be applied, how frequently they should be used, or how specific thresholds should influence clinical decision-making. Even in studies exploring differential diagnosis across PSP phenotypes or distinguishing PSP from other conditions [e.g., Ugga et al., (2020)], MRPI-based measures are described primarily as supportive tools at the stage of initial evaluation, without a defined clinical workflow.

Across the available evidence, a consistent pattern emerges. MRPI and related planimetric indices are most useful in patients with clinically uncertain or early-stage parkinsonism, particularly when differentiation between PSP, Parkinson’s disease, and, to a lesser extent, MSA is challenging. In this context, they increase diagnostic confidence but do not replace clinical criteria. As highlighted in studies by Grijalva et al. (2022); Brinia et al. (2023) their diagnostic performance is highest in typical PSP-Richardson’s syndrome, while sensitivity may be lower in variant phenotypes.

Automated volumetric approaches extend this framework by enabling multiregional assessment of disease-related atrophy. As shown in machine learning based analyses by Quattrone et al. (2022) these methods may further improve diagnostic classification and support prognostic stratification. However, their application remains largely limited to research settings and specialized centers due to technical complexity, lack of standardization, and variability across MRI acquisition protocols.

Importantly, a recurring theme across all cited studies is the gap between methodological development and clinical implementation. While morphometric MRI markers are often described as “clinically applicable,” explicit guidance on their integration into routine workflows is rarely provided. As a result, these techniques are not yet routinely incorporated into everyday clinical decision making.

Beyond cross sectional discrimination, volumetric MRI has gained importance as a potential progression biomarker and outcome measure. Longitudinal volumetry can capture disease related volume loss over relatively short intervals and may increase statistical power in interventional studies, particularly when advanced longitudinal post processing strategies are used to reduce variability and permit centralized image reading (Krismer et al., 2024).

### 7.2 Methodological and practical limitations

Nevertheless, several practical limitations still constrain broader clinical implementation. A key challenge is heterogeneity of MRI acquisition protocols (field strength, sequence parameters, scanner differences, upgrades), which can introduce systematic variability in volumetric measures. For example, in a large multicenter atlas-based study, acquisition relied on routine clinical MPRAGE sequences at each center without harmonization, underscoring the real world nature of the data but also highlighting protocol driven variability as a major confounder in volumetric studies (Huppertz et al., 2016).

A substantial body of evidence demonstrates that quantitative MRI measures are sensitive to scanner- and protocol-related factors. Han et al. (2006) showed that cortical thickness measurements vary depending on field strength, scanner manufacturer, and pulse sequence, even under otherwise controlled conditions. Similarly, Jovicich et al. (2009) demonstrated that while within-scanner reproducibility is high, combining data across scanners, vendors, and field strengths introduces systematic bias that may affect volumetric estimates.

This issue becomes even more pronounced in multicenter settings. In a large ADNI-based analysis, Kruggel et al., (2010) reported that variability of volumetric measures can increase up to tenfold when different acquisition conditions are used in the same subject, highlighting the critical impact of scanner-dependent factors on measurement precision.

More broadly, Fortin et al. (2018) introduced the concept of “scanner effects,” emphasizing that non-biological variability can obscure true disease-related differences and lead to spurious findings if not properly addressed.

Recent large-scale studies further underscore the importance of harmonization. Pomponio et al. (2020) analyzing over 10,000 MRI scans across multiple cohorts, demonstrated that pooling data from different sites without harmonization significantly limits interpretability, whereas appropriate harmonization strategies improve reproducibility and enable meaningful cross-cohort comparisons. In parallel, methodological advances in machine learning–based harmonization, such as the approach proposed by Zuo et al. (2021) aim to reduce inter-site contrast variability while preserving anatomical information, further supporting the need for standardized processing frameworks.

Taken together, these findings highlight that scanner-related variability is not a marginal issue but a central challenge for the clinical translation of MRI volumetry.

In addition to technical variability, quantitative analyses are sensitive to biological and nuisance covariates, most notably age and head size (total intracranial volume, TIV/ICV). Empirical studies in healthy populations demonstrate that these factors explain a substantial proportion of variance in regional brain volumes. Importantly, simple normalization approaches, such as dividing by TIV, are often insufficient, as regional volumes do not scale proportionally with head size. Scanner upgrades may further influence thickness and volume measures, complicating longitudinal and cross-sectional comparisons.

At the level of simpler planimetric indices, ratios (e.g., midbrain-to-pons) partially mitigate head size effects and may yield excellent interrater reliability, supporting feasibility in clinical practice; however, these approaches still depend on consistent slice selection and measurement conventions and do not replace comprehensive whole brain volumetry (Massey et al., 2013).

Methodologically, voxel based and atlas based approaches rely on accurate segmentation and spatial normalization, which may be affected by image quality, atrophy severity, and algorithmic assumptions. Early VBM methodology emphasizes that volumetric inference depends on robust tissue classification and normalization pipelines, which can vary across software versions and processing settings (Sjöström et al., 2020).

Finally, routine clinical deployment is limited by the availability of computational infrastructure, trained personnel, and standardized normative datasets. Although large-scale automated pipelines are increasingly feasible, quality control remains essential, and lack of harmonized normative references (age-, sex-, and scanner-specific) continues to limit individual-level interpretation in everyday reporting.

### 7.3 Future perspectives

Future developments in MRI volumetry are expected to further strengthen its clinical utility in atypical parkinsonian syndromes. Key priorities include standardization of acquisition protocols and post processing pipelines, particularly in multicenter settings, to reduce variability and improve comparability across studies and clinical sites. The establishment of large, harmonized normative datasets stratified by age, sex, and scanner characteristics will be essential to support reliable individual level interpretation.

Integration of automated volumetry with machine learning models may further enhance diagnostic accuracy, particularly in early disease stages. However, emphasis should be placed on model interpretability, external validation, and robustness across scanners and populations to ensure clinical trust and regulatory acceptance. Hybrid approaches combining targeted indices (e.g., MRPI 2.0) with multiregional automated volumetry may represent a pragmatic balance between feasibility and diagnostic performance.

With specific regard to progressive supranuclear palsy, future diagnostic strategies may benefit from a longitudinal, pathway oriented volumetric approach that captures the temporal evolution of neurodegeneration rather than relying solely on static cross sectional markers. Increasing evidence suggests that PSP pathology follows a characteristic spatiotemporal progression, initially involving the midbrain and related brainstem structures and subsequently extending to cerebellar, thalamic, and cortical regions (Kumar et al., 2025). Volumetric assessment of this evolving pattern over time may improve early phenotypic differentiation, particularly in non Richardson PSP variants in which classical brainstem markers are less pronounced at disease onset.

Within this conceptual framework, MRPI 2.0 may be regarded as a quantitative imaging marker of the early stage of the PSP disease trajectory. By incorporating ventricular measurements in addition to classical brainstem parameters, MRPI 2.0 appears well suited to capture early periventricular and diencephalic involvement that may precede or accompany overt midbrain atrophy. In this context, MRPI 2.0 should be viewed not merely as a refined cross-sectional discriminator, but as an anchor marker reflecting an early step along the spatiotemporal pathway of PSP related neurodegeneration.

Longitudinal application of MRPI 2.0 may therefore allow tracking of disease progression along this trajectory, particularly in non Richardson PSP phenotypes in which static midbrain based indices show limited sensitivity at symptom onset. When integrated with whole brain volumetric analyses, MRPI 2.0 may serve as a central reference point for pathway oriented assessment, linking early ventricular and brainstem changes with subsequent involvement of cerebellar, thalamic, and cortical regions.

Beyond their role in monitoring disease progression, there is growing interest in the potential of MRI-based measures to act as prognostic biomarkers, i.e., to predict future clinical trajectory. In multiple system atrophy, structural MRI abnormalities at baseline have been associated with faster subsequent clinical deterioration, as shown by Krismer et al. (2019) suggesting that imaging-defined subgroups may differ in disease aggressiveness. Similar observations have been reported in PSP, where midbrain atrophy, MRPI, and ventricular enlargement have been linked to clinically meaningful outcomes, including earlier loss of ambulation and more rapid functional decline (Picillo et al., 2020). Furthermore, structural MRI correlates of survival have been identified, with regional atrophy in striatal, cerebellar, and frontotemporal regions associated with shorter survival, supporting a role for imaging in prognostic stratification (Street et al., 2023).

At the same time, recent large-scale analyses indicate that the prognostic value of MRI should be interpreted with caution. In a multicohort study, Quattrone et al. (2025) showed that although certain regional volumetric measures are associated with disease progression at the group level, they have limited ability to accurately predict individual trajectories, particularly when tested using machine learning models. This highlights an important distinction between group-level biomarkers and clinically actionable prognostic tools.

Taken together, these findings suggest that MRI volumetry holds promise not only as a marker of disease progression but also as a potential component of prognostic assessment. However, its predictive value remains context dependent and currently insufficient for individual level prediction. Future work should therefore focus on integrating imaging markers with clinical, molecular, and possibly multimodal biomarkers to improve prognostic accuracy and support personalized approaches to disease management.

## 8 Conclusion

MRI-based morphometric approaches have emerged as a powerful and clinically relevant extension of conventional neuroradiological assessment in atypical parkinsonism. Disease specific volumetric patterns midbrain atrophy in PSP, ponto-cerebellar and putaminal involvement in MSA, and asymmetric cortical degeneration in CBS provide objective markers that enhance diagnostic confidence and support differential diagnosis. Advances in atlas based segmentation and automated analysis have further expanded the scope of volumetric MRI, enabling comprehensive, multiregional assessment of neurodegeneration.

Despite current limitations related to acquisition variability, normalization strategies, and access to advanced analysis tools, quantitative MRI holds substantial promise for routine clinical practice and research applications. In addition to its established diagnostic role, growing evidence supports its use as a marker of disease progression and, potentially, as a component of prognostic assessment. However, its predictive value at the individual level remains limited, highlighting the need for further validation and integration with clinical and multimodal data. With continued methodological refinement, MRI-based morphometry is well positioned to contribute to earlier diagnosis, improved phenotypic characterization, and the development of robust biomarkers for future therapeutic trials in atypical parkinsonian syndromes.
